# How does the leniency of personal bankruptcy law affect entrepreneurship in EU countries?

**DOI:** 10.1371/journal.pone.0272025

**Published:** 2022-07-28

**Authors:** György Walter, Ferenc Illés, Fanni Tóth

**Affiliations:** Department of Finance, Corvinus University of Budapest, Budapest, Hungary; Szechenyi Istvan University: Szechenyi Istvan Egyetem, HUNGARY

## Abstract

Several studies examined how some characteristics of personal bankruptcy laws influenced entrepreneurial developments during the last two decades. Our main objective is to analyze the association between self-employment and the leniency of the personal bankruptcy systems in 24 EU countries. Unlike previous studies, we measure differences and changes in the leniency of the regulations with a composite index that incorporates 35 variables. Based on a cross-country database of self-employment ratios and various control variables spanning the years 2000 to 2019, we apply a panel regression model. We find that the implementation of new regulations and reforms in personal bankruptcy legislation in more lenient directions positively correlates with entrepreneurial developments measured by self-employment rates. This is more significant in the group of countries where the eligibility criteria for entrepreneurs are not constrained. We find a one-year negative time-lag effect and conclude that strong anticipation of the law for a more lenient system can immediately change the risk-reward profile, and thereby influence entrepreneurship before implementing the actual reform. An important policy implication is that a major reform in regulation or the first implementation of conservative legislation has the same order of magnitude of effect on promoting entrepreneurship as other public policy reforms of similar purpose.

## Introduction

The perception of the importance of entrepreneurship has changed significantly in recent decades among academics and public policy makers. In the 1950s and 1960s, the importance of entrepreneurship declined. The typical view was that it might be important from a social and political point of view but not relevant from an economic perspective due to efficiency and economy of scale. A reverse thinking approach had emerged in the 1970s and 1980s, as empirical studies started to underspin the reemergence of entrepreneurship, shifting the focus on the input factors of innovation and ideas rather than natural resources and capital. From the 1990s, the role of entrepreneurship has also been the key discussion point for policy makers. [1–3]; In the last two decades, several empirical studies have analyzed the impact of entrepreneurship on economic, social, and environmental welfare. Besides economic impacts, the latest research also considered social and environmental effects. Neumann [4] concluded that most empirical research in the last 25 years showed that entrepreneurship had a significant positive impact on macroeconomy, social welfare, and found mixed evidence on environmental impact. In the last 10–15 years, the spread of alternative work arrangements and the opportunity of platform work has also been strengthening the policy implication and importance of self-employment and entrepreneurship [5, 6].

There are several opportunities for policy makers to influence entrepreneurship by changing different regulations. Besides relevant regulatory changes in the tax system, the labor market, or subsidies, an opportunity for policymakers to influence the risk-reward profile of potential investors and thus entrepreneurship is to modify the leniency level of personal bankruptcy legislation [2, p.31]. The leniency of a personal bankruptcy system is a synthesizing term; it shows “how the system handles the defaults of private individuals and entrepreneurs with unlimited liabilities, how easy or difficult it is for borrowers to achieve a fresh start, and additionally, how harsh or lenient various possible stigmas are after receiving a fresh start” [7, p.2]. Personal bankruptcy systems and their leniency elements–such as conditions for a fresh start, conditions of discharge, exemptions–have become crucial factors for policymakers and researchers to analyze the impact of differences or changes in leniency on the level of entrepreneurship cross-time or cross-countries (among the US-federal states). These studies represent a large group of recently published studies in the field of personal bankruptcy. However, these studies were typically limited to focus on the impact of either 1) a one-time event of a specific legislative change, typically big reforms of systems in the US or Europe; or 2) regional differences in some selective elements, like homestead exemptions, judicial customs of the US federal system, or some selected discharge-variables of the legislation of the selected countries. The research gap to create a complex measure of leniency was filled by Walter and Krenchel [7] who created a composite index of personal bankruptcy legislation and measured and compared the levels of leniency of 25 EU countries and the US as a benchmark for 2020. The development of the leniency index and the validation of the countries’ leniency levels created an opportunity to fill an additional gap in the entrepreneurial literature: to analyze the association between personal bankruptcy systems reforms and entrepreneurship cross-time and cross-country.

Our research aims to analyze the association between entrepreneurship (measured by self-employment developments) and the leniency level of the personal bankruptcy systems (measured by 35 variables describing debtor friendliness) in all EU countries. The purpose is to detect whether there is any significant change in entrepreneurial development if a personal bankruptcy legislation is accepted or reformed in the EU between 2000 and 2020.

As a starting point, we take the results of the Walter and Krenchel leniency index [7] for 2020, then we determine and measure the changes in the indices of the EU countries from 2000 to 2019. We examine the association of changes in leniency levels and changes in self-employment data with a panel regression. Our aim is to detect an association between leniency changes and entrepreneurship and to compare our results with the findings of other empirical studies. We also aim to provide a tool for policymakers to understand how and to what extent changes in the different elements of the personal bankruptcy legislation can influence self-employment and to compare it to other possible policy measures discussed in the literature.

In the following sections, we first show the literature that focuses on the association between personal bankruptcy and entrepreneurship. Then we measure and determine the changes and development of the leniency indices of the EU countries from 2000 to 2019. In the next section, we present the details of the panel regression, data, methodology, and results. We discuss the main conclusions in the final section.

## Review of the literature

The literature of the past decades agrees on the importance of entrepreneurship in politics, the economy, society, and recently environmental concerns. Numerous studies that examined the impact of entrepreneurship on economic welfare not only showed positive effects on GDP measures or employment, but also on innovativeness or competitiveness. These studies analyzed the impact of environmental, individual, and firm-level determinants. The empirical studies of the last 25 years were systematically reviewed by Neumann [4].

On the other side of the coin, several studies analyzed the key factors determining the development of entrepreneurship. These determinants in the literature range from economic to historical, psychological, social, cultural, and political factors. Audretsch [2] discussed supply and demand factors, macro and micro factors that can be analyzed at an individual level (income choices, unemployment, earnings differentials, value independence) and firm-level (entry barriers such as advertising intensity, R&D and capital requirements). The study presented the roles of the following key factors: access to finance, administrative burdens and taxes, immigration, female entrepreneurship, geography, spatial location, culture and social capital. Hofstede et al. [3] focused on the determinants of entrepreneurship at the country level to clarify cross-country differences. They presented traditional economic explanations such as prosperity (shown by per capita income or GDP), unemployment rate, tax rates, female labor share, earning differentials, population density, etc. They also pointed to the role of cultural traits such as individualism, uncertainty avoidance, and dissatisfaction. After summarizing previous empirical studies, Parker and Robson [8] compared self-employment rates in developed countries, and the most mentioned determinants included per capita GDP, unemployment, female labor force participation and tax rates. Armour and Cumming [9] also focused on economic determinants of entrepreneurship such as taxation, protection of property rights, labor market regulation, the financial cost of incorporating a business, the availability of finance, but specifically on personal bankruptcy law, which allows a fresh start and acts as social insurance.

In his detailed literature survey, Audretsch [2, p.31] provided a framework for these determinants of entrepreneurship and defined the different types of entrepreneurship policies: 1) shaping opportunities and demand, like deregulation of market entry, privatization of sectors, access to government programs, etc.; 2) shaping supply side, such as facilitating excluded minorities, enhancing skills, providing microcredit; and 3) changing the risk-reward profile directly via taxes, subsidies, labor market rules, and bankruptcy regulation. There were also empirical studies that analyzed the magnitude of the impact of selected policies and factors on entrepreneurship. For example, Heim and Lurie [10] found evidence on the association between deductibility of health insurance premiums and the probability of becoming self-employed after analyzing US data from 1999–2004. Besides the positive correlation, they concluded that the magnitude of the effect was around a 0.3–1.5% increase in the probability. Jha and Bhuyan [11] searched for an explanation of whether financial reforms promote entrepreneurship by comparing data from 41 countries. Financial reforms were measured by a composite index called the “Financial liberalisation index”. They found a significant positive association between the overall financial sector reforms and specifically with the dimensions of reforms in directed credit, credit controls, banking supervision, and international capital flows with early-stage entrepreneurial activity. The magnitude of having a general reform that increases the “Financial liberalisation index” by 1 resulted in an increase in entrepreneurship by 1.38% [12].

It is an important question in all relevant empirical studies, that is, what to use as proxy measures for the intensity and the magnitude of entrepreneurship. Commonly used proxy methods are self-employment rates, business ownership rates, new-firm start-ups and formations, and other measures of industry demography like the extent of simultaneous births and exits, net entry and foundation indices [2, 4]. Self-employment is the most used proxy as it is regularly measured, highly available, and comparable in most countries [13]. Self-employment is also frequently used to study the relation between personal bankruptcy and entrepreneurship. The studies of Fan and White [14], Armour and Cumming [9], Akyol and Athreya [15], Mankart and Rodano [16], Primo and Green [17] discussed in the following paragraphs also used self-employment in their studies when they analyzed the association between differences in personal bankruptcy systems and their impact on entrepreneurship.

A significant part of the literature on personal bankruptcy systems focused on understanding how changes and differences in personal bankruptcy systems affected entrepreneurship. If protection is reduced, there are opposing effects on the market. Firstly, debtors lose some of their existing insurance which assumably negatively influences entrepreneurship. On the other hand, due to “credit rational” effects, creditors can lower interest rates, and expand credit demand that allows new debtors to enter the market [18].

Several studies underpin the existence of the expected credit rational in the case of moving to more lenient legislation. Gropp et al. [19] found that bankruptcy exemptions redistributed credit from low-asset households toward borrowers with high assets. They concluded that there was more credit rationing in states with more lenient regulation as debtors were more likely to default and file for bankruptcy. Lin and White [20] created a model based on a data set from the mid-1990s to analyze the relation between high exemption and credit rationing in the home purchase and home improvement mortgage loan market. They concluded that high exemption should have resulted in more credit rationing as lenders face additional default costs in filing cases. White [21] examined the effect of the BAPCPA on household debts and found that conservative reform resulted in a significant increase in the volume of revolving household debts. Davydenko and Franks [22] examined the firm data of some European countries (France, Germany, and the UK) and analyzed the bankruptcy laws and their relation to the credit markets. Their results indicated that banks responded to more lenient legislation with stricter collateral requirements. Han and Li [23] focused on household borrowing after closing bankruptcy. They found that filers had more limited access to unsecured credit. Lilienfeld and Toal-Mookherjee [24] showed in a two-sided matching model that changing exemption levels in bankruptcy law induced a redistribution of credit. A more lenient system resulted in that lending to poorer borrowers shrinks, while for the richest agents grew and the cost of borrowing shrank. Cerqueiro and Penas [25] supported these later findings and empirically analyzed the effect of debtor protection by differentiating three levels of wealth.

Some papers conflicted the results of the positive relationship between leniency in the personal bankruptcy regulation and the appearance of credit rationing. Berkowitz and Hynes [26] argued that due to a positive wealth effect, financially distressed homeowners filing for bankruptcy would be able to retain more of their assets in high-exemption states, which would enable them to continue paying their mortgages. Alexandrov and Jimenez [18] examined the effect of BAPCPA on the student loan market examining different credit score classes. They concluded that BAPCPA did not have a significant effect on the price of loans for the lowest credit score individuals relative to students with higher credit scores. Simkovic [27] analyzed the change in credit conditions for consumers due to the introduction of BAPCPA and found little for improvement. Hintermaier and Koeniger [28] also compared homestead exemptions in the US regulations and found, that bankruptcy regulation showed a limited effect on the quantity and price of unsecured debt.

On the other hand, the relevant literature also analyzed the intensity of the insurance effect of the leniency elements and the related entrepreneurial incentives. The studies focusing on the effect of fresh start and leniency on entrepreneurial activity typically investigated whether more debtor-friendly personal bankruptcy systems (differences in exemption levels) or changes in the legislation (reforms) had an overall positive effect on entrepreneurial activity. Fan and White [14] examined state-level exemptions across the US states and their effect on self-employment. Based on their findings, the greater state-level exemptions were associated with an increase in entrepreneurship and the probability of starting self-employment was significantly higher in those states where exemptions were unlimited rather than low. Agarwal et al. [29] analyzed the relation of small business owners filing to homestead and personal property exemption levels in various US states. They found a positive correlation between the likelihood of filing and the exemption levels. Primo and Green [17] also focused on the association between home exemption levels in the US and self-employment. They concluded that more refined measures of entrepreneurship were needed as results showed that more generous law led to a lower level of “innovative” entrepreneurship. Landier [30] concluded that lenient bankruptcy rules encouraged entrepreneurs to experiment, as they made it easier to close an unsuccessful business and have a fresh start. Armour and Cumming [9] focused on 15 countries in North America and Europe with the 1990–2005 data set. With a panel regression analysis, they measured the leniency of bankruptcy laws using five bankruptcy indices (variables), such as the availability of discharge, the length of discharge, the minimum capital to form a company, the exemptions, the potential disabilities, and the conditions of discharge. They found that leniency significantly increased self-employment rates. Peng et al. [31] analyzed the database of 25 countries on different continents and compared bankruptcy laws based on six dimensions, highlighting that entrepreneur-friendly bankruptcy laws could lower entry barriers for entrepreneurs. With a different approach, Mankart and Rodano [16], presented a general equilibrium model and finally concluded that a more lenient bankruptcy law in the US would increase entrepreneurial activity in the country. Lee et al. [32] used the basic methodology of Armour and Cumming [9] and took over the leniency-dimensions of Peng et al. [31], Claessens and Klupper [33] and the creditor protection variables of La Porta et al. [34] and examined the database of 29 countries from 1990–2008. In their panel regression, they examined the correlation between lenient and entrepreneur-friendly corporate bankruptcy laws and entrepreneurial developments. The leniency of the systems was measured by five observed variables of the bankruptcy procedures: time spent on bankruptcy, cost of bankruptcy, recovery rate at a fresh start, and two dummy variables of having control of assets and staying of incumbent management. They found a significantly positive correlation with all variables describing leniency. Estrin et al. [35] analyzed 15 developed countries and the impact of some elements of bankruptcy codes on entrepreneurial activity with prospect theory and found that asset protection played one of the most significant roles. Finally, Lee et al. [36] presented a real options perspective to show how entrepreneur-friendly bankruptcy encouraged entrepreneurship developments.

Some articles conflicted with the results of the positive entrepreneurial effects of lenient systems. Meh and Terajima [37] elaborated a quantitative overlapping-generations model with the conclusion that eliminating bankruptcy exemptions would lead to a modest increase in the fraction of entrepreneurs. Cumming and Li [38] examined business births and deaths during 1995–2010 in the United States, focusing on homestead exemptions and entrepreneurial development. The study showed a positive impact only among the states in the bottom quartile; otherwise, it demonstrated a negative impact. Albanesi and Nosal [39] analyzed the effect of the BAPCPA reform, finding a negative effect of strict measures in the bankruptcy regulation on entrepreneurship. They showed that the main effect of transforming the system into a less lenient one resulted in a shift among financially distressed individuals from straight bankruptcy to insolvency. They did not find evidence of an increase in the propensity to remain solvent or to cure debt. They also showed that insolvency was associated with a high degree of financial distress compared to bankruptcy. Fossen and König [40] analyzed the German reform of 2014, where the compliance period was reduced, which should have served as an incentive for more people to start entrepreneurship. However, they could not produce notable results to justify their hypothesis. Patel and Devaraj [41] also examined the US state-level exemptions, their changes in legislation, and their effect on entrepreneurial activity. They concluded that entrepreneurial activity did not improve due to asset protection in personal bankruptcy systems.

Some of the papers reported more mixed findings on the net outcome of credit rational and the insurance effect on entrepreneurship. Georgellis and Wall (2006) [42] found that in the case of small changes in exemptions, the credit supply effect was important, however for larger changes, the insurance effect dominated. Jia (2015) [43] compared several systems of different countries and separated classes based on entrepreneurial ability. As a result, there was an inverted U-relationship between the asset exemption level and the level of entrepreneurship in the US. By moderate-ability households the borrowing effect denominated, therefore they preferred less lenient systems. On the contrary, classes with the highest entrepreneurial ability preferred a more lenient system.

Finally, besides entrepreneurial developments, some papers discussed the association between labor incentives and the forgiveness of personal bankruptcy laws. Han and Li [44] analyzed the fresh start policy, not from a business entrepreneur perspective, but whether discharge preserved human capital by maintaining incentives to work. They found–however the effects were not statistically significant–that due to the wealth effects, the filing for bankruptcy did not have a positive impact on annual work hours for bankrupt households. Chatterjee and Gordon [45] examined a no-bankruptcy world and like Han and Li [44], they concluded that the bankruptcy option did not result in a superior labor supply incentive. Dobbie and Song [46] criticized the results of Han and Li [44] explaining why studies had found little evidence about the positive effect of protection for debtors.

In summary, the papers listed above typically examined the effect of leniency on the credit market or entrepreneurial developments by focusing on homestead exemptions among the US states or analyzing the effects of one-time reforms. Some studies examined the association between entrepreneurial developments and bankruptcy systems of selected developed countries on different continents. These articles typically characterized creditor friendliness by a few selective variables (focusing on discharge element or asset protection) and calculated panel regression. Many articles found positive associations; however, some papers contradicted these results.

Although the EU countries have very heterogeneous personal bankruptcy systems [7], no extensive research on the association between leniency and entrepreneurial development in the whole EU has been done. Previous studies did not incorporate complex bankruptcy variables varying in time. Like other articles [9, 32], we also focus on the association between the leniency variables of bankruptcy systems and entrepreneurship. However, filling the literature gap, we measure the level of leniency and the changes in these systems with a group of 35 different time-varying variables that cover all important dimensions of leniency. We measure these variables and their changes and examine the association in all EU countries that introduced personal bankruptcy systems. Our hypothesis is that by measuring the leniency with a time-varying composite index, we find a positive association between self-employment rates and the leniency index of the very heterogeneous systems in the EU. In the next section, we present the data, methodology, and empirical specifications of the research.

## Data and methodology

The purpose of this research is to isolate the ceteris paribus effect of the personal bankruptcy system on self-employment. Regulation varies across counties; however, we are not interested in country-specific effects, but rather the general association. Additionally, the personal bankruptcy law changes over time. Therefore, we need to analyze a combination of cross-sectional and time-series data. We have to isolate the effect of the personal bankruptcy system from the time-invariant country-specific effects and time-varying variables that can also affect our dependent variable and may cause endogeneity.

Our research scope covers all 27 EU countries. As Malta and Bulgaria do not have personal bankruptcy systems, these countries are not examined. Most of the necessary self-employment data and control variables for Cyprus are not available or not comparable; therefore, we also excluded it from the calculation. Thus, we study data on self-employment and bankruptcy law over the years 2000–2019 in 24 EU countries: Austria, Belgium, Denmark, Finland, France, Germany, Greece, Ireland, Italy, Sweden, the Netherlands, Luxembourg, Poland, Spain, Estonia, Portugal, Slovakia, Slovenia, Czech Republic, Latvia, Lithuania, Hungary, Croatia, and Romania.

As we have 2-dimensional data, across countries and time, we assume, consistent with studies with similar research focus [9, 32, 47], that the data-generating process is a panel regression model described by the equation:

$$y_{i,j}=\beta_{0}∙L_{i,t}+\sum_{j}\beta_{j}∙X_{i,t}^{\left(j\right)}+a_{i}+\varepsilon_{i,t}$$

where *i* and *t* denote country and year, respectively, *L* is our leniency index, the *X*^*j*^s are our control variables including time dummies *ε*_*i*,*t*_ is the error term, *y*_*i*,*t*_ is the dependent variable, and *a*_*i*_ contains all time-invariant country-specific effects. From 2000 to 2019 self-employment cannot be considered as a stationary variable. In several countries, there is a clear trend in time (S1–S3 Figs), therefore our dependent variable is the change in self-employment based on the definition of the World Bank that serves also as the data source: “Self-employed, total (% of total employment, modelled ILO estimate)”, see definition and source in S1 Table. We employ the fixed effects estimator to the *β*_*j*_ coefficients in the equation. It is not our intention to predict self-employment, but to test the marginal effect of the leniency proxy variable, so we are primarily interested in the sign and significance of its coefficient [48].

**Fig 1:**
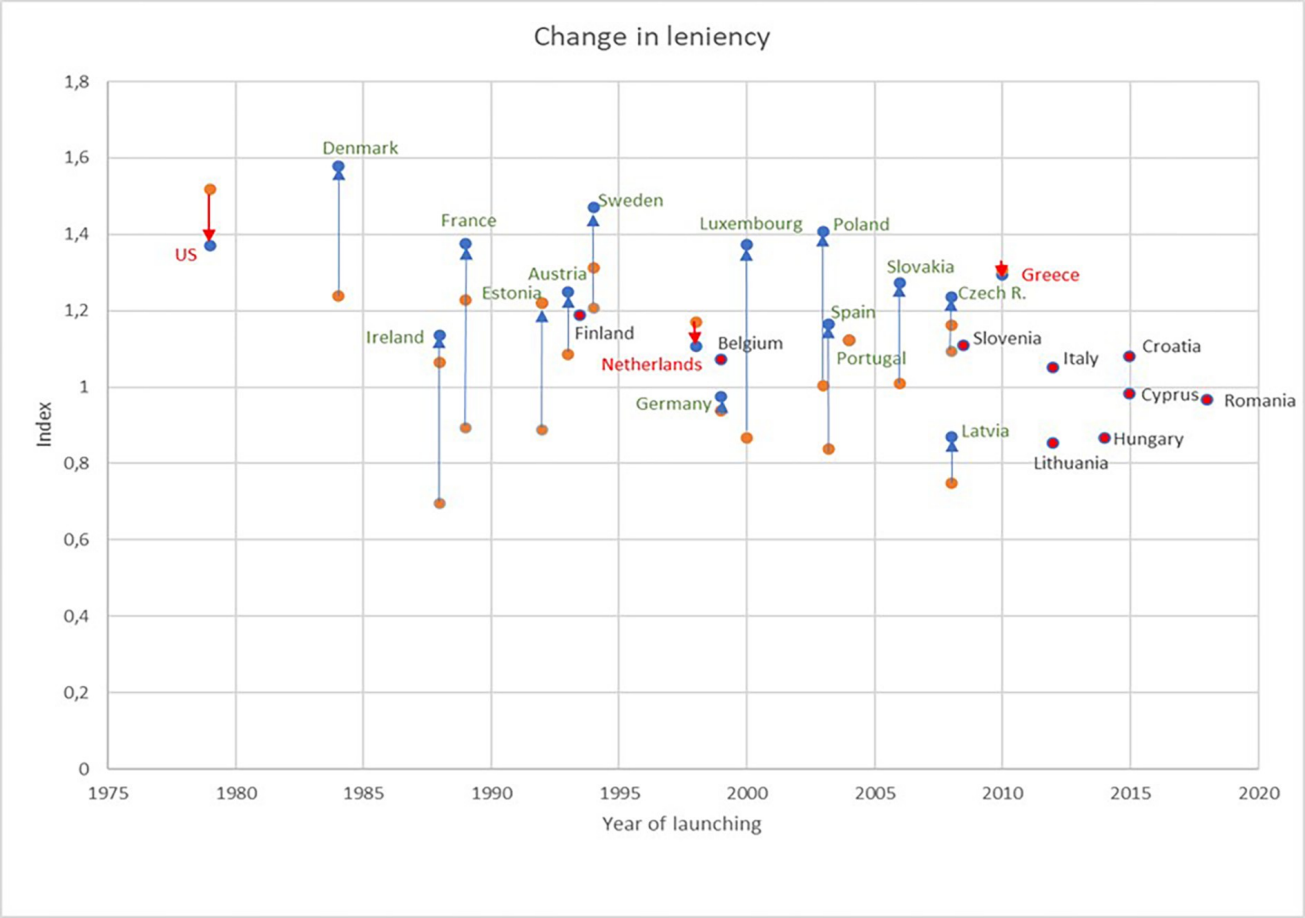
Leniency level of the 24 EU countries and the US in 2020 and cross-time. Axis “year of launching” presents the year when the first legislation came into effect. Arrows show the change in leniency in case of one or two major reforms, if any. Two countries with less-lenient reforms are marked in red. Countries marked in red points without arrows indicate that no major reforms have yet been implemented.

We examine the changes in legislation from 2000 to 2020 of all countries, the rescored variables, and the dimensions of the index, and finally evaluate the changes in the composite indices of the 24 countries. Data sources for the examination include the countries’ local legislations, laws and the excessive study and book of Graziano et al. [49] (See the country reports of Melcher and Lurger (Austria), Storme and Helsen (Belgium), Garasic (Croatia), Sprinz (Czech R.), Orgaard (Denmark), Sajadova and Viirsalu (Estonia), Jaatinen and Remes (Finland), Rublellin and Booth (France), Keinert and Vallender (Germany), Venieris (Greece), Holohan and Farry (Ireland), Cerini et al. (Italy), Sajadova (Lithuania), Hoffeld and Franczak (Luxembourg), Jungmann and Madern (The Netherland), Porzicky and Rachwal (Poland), Carvalho et al. (Portugal), Zidaru (Romania), Orsula (Slovakia), Dordevic (Slovenia), Arias (Spain), and Hellström (Sweden) as given in [49]) The index scores are between 0.0 and 2.0, where the 0.0 score represents that there is no personal bankruptcy system in the country that specific year. A higher score on the index indicates a higher level of leniency of the country. (See S2 Table for the description of the variables in the leniency index.) Fig 1 illustrates the cross-country and longitudinal data set of the leniency index, also presenting the United States as a benchmark. As can be assumed based on the results of Walter and Krenchel [7], all reforms were made in a lenient direction except for two cases (Greece and the Netherlands). Countries that have not materially changed the legislation since the start typically launched their systems after 2010 and usually started at a low-lenient level.

It is not our intention to create a predictive model; our aim is to include the variables that are observable and correlate with those included in the model, as the uncorrelated variables could increase *R*^*2*^ but do not change the beta coefficient and its significance. Therefore, we control for several economic variables based on the literature on entrepreneurship and personal bankruptcy [3, 8, 9, 32]. We control for countries’ general levels of development, performance, and macroeconomic variables such as “GDP growth rate”, gross domestic expenditures on research and development (“R&D”), “market index returns” and their “volatility”. We control labor market variables such as “unemployment rate”, “female labor force participation”, and “personal income tax rates”. We also control for the social-economic factor of inequality in income distribution (“Gini index”). Definitions and sources of these variables are presented in S1 Table. We employ a fixed-effects estimator and assume that culture tends to be invariable during the examined period, and therefore the omission of cultural factors is not a concern [11].

We control for time effects across all countries. We include a variable that measures years elapsed from 2000 to capture any time trend effects associated with changes in the bankruptcy rate [32].

The summary statistics of the variables of the 24 countries are reported in Table 1.

**Table 1:** Summary statistics of variables.

Variables	N	Minimum	Maximum	Mean	Std. Deviation
Self-employment_Diff (dependent)					
Austria	19	-0.0053	0.0064	-0.0007	0.0032
Belgium	19	-0.0082	0.0073	-0.0010	0.0045
Croatia	19	-0.0246	0.0098	-0.0061	0.0111
Czech Republic	19	-0.0092	0.0153	0.0009	0.0058
Denmark	19	-0.0051	0.0049	-0.0004	0.0025
Estonia	19	-0.0169	0.0166	0.0014	0.0079
Finland	19	-0.0085	0.0081	0.0000	0.0038
France	19	-0.0051	0.0072	0.0004	0.0037
Germany	19	-0.0037	0.0070	-0.0007	0.0028
Greece	19	-0.0236	0.0061	-0.0053	0.0087
Hungary	19	-0.0097	0.0112	-0.0023	0.0049
Ireland	19	-0.0088	0.0073	-0.0025	0.0045
Italy	19	-0.0124	0.0031	-0.0032	0.0032
Latvia	19	-0.0204	0.0127	-0.0017	0.0079
Lithuania	19	-0.0289	0.0072	-0.0042	0.0108
Luxembourg	19	-0.0126	0.0201	0.0003	0.0067
Netherlands	19	-0.0026	0.0157	0.0028	0.0041
Poland	19	-0.0132	0.0057	-0.0039	0.0048
Portugal	19	-0.0219	0.0182	-0.0049	0.0087
Romania	19	-0.0632	0.0239	-0.0115	0.0199
Slovakia	19	-0.0048	0.0260	0.0038	0.0078
Slovenia	19	-0.0231	0.0207	-0.0013	0.0140
Spain	19	-0.0108	0.0091	-0.0023	0.0044
Sweden	19	-0.0053	0.0028	-0.0007	0.0023
Leniency index	456	0.0000	1.5800	0.8415	0.5026
Control variables					
Unemployment	418	0.0181	0.2747	0.0896	0.0455
R&D	431	0.0036	0.0391	0.0158	0.0088
FemLaborForce	456	0.3590	0.6119	0.5105	0.0538
GDP growth rate	456	-0.1484	0.2516	0.0233	0.0353
Gini	374	0.2370	0.3960	0.3123	0.0368
TaxRate	399	0.1460	0.4296	0.2865	0.0573
IndexReturn	377	-1.2125	0.9452	0.0280	0.2864
IndexVolatility	385	0.0206	0.9530	0.1774	0.1019

Our data pool for the panel regression consists of 244 observations in total (“Total Group”) for the years 2000–2019. We form a subgroup (“Adjusted Group”) of 172 observations, where we exclude four countries: Belgium, France, Luxembourg, and Romania. The systems of these countries allow only limited eligibility for entrepreneurs to participate in the process; it is restricted to whether liabilities from business or commercial activity are eligible for the personal bankruptcy process. These distinctions in eligibility could highly influence incentives for starting entrepreneurship, and thus the association between the leniency of the system and entrepreneurship. This approach differs from other studies using leniency variables for panel regressions.

*T* indicates the year in which the legislation entered into force. We also repeat our calculations based on different time lags. To examine the effect of whether the reaction of potential entrepreneurs shows a delay, in addition to running the calculation of *T*, we also calculate our results with a time-lag of plus one year (*T+1*). Since parliamentary debates and the process of passing legislation may last several years, we also look at whether the population might react to the anticipated changes before they come into force. Therefore, we also checked our calculation for the year before entering into force of the legislation (*T–1*). Finally, to verify robustness, we also run our calculation for (*T–2*).

## Results

Our main results are reported in Tables 2 and 3. Table 2 presents the results of the total group of 24 countries with different time-lags (+1, 0, –1 year). The level of leniency of personal bankruptcy systems is positively and significantly associated with the change in self-employment rates in the case of no time-lag (*T*) and, somewhat surprisingly, with a negative time-lag of one year (*T–1*). However, we find a less significant association in the case of a positive time-lag of one year (*T+1*).

**Table 2:** The leniency of personal bankruptcy and self-employment: total group with different time lags.

	Total Group		Total Group		Total Group	
	(N = 244, *R*^2^ = 21%)		(N = 244, *R*^2^ = 21%)		(N = 244, *R*^2^ = 20%)	
	Time lag: -1		Time lag: 0		Time lag: +1	
	Beta	p value	Beta	p value	Beta	p value
Leniency index	0.00422**	1.05%	0.00396**	1.27%	0.00301*	5.80%
Unemployment rate	-0.0084	58.72%	-0.01038	50.78%	-0.00993	53.40%
R&D	-0.1013	60.83%	-0.14172	47.61%	-0.14981	45.75%
FemLaborForce	0.048	10.88%	0.05114*	8.63%	0.05544*	6.39%
GDP_growth_rate	-0.0496***	0.30%	-0.04746***	0.47%	-0.04939***	0.34%
Gini Index	-0.076**	2.65%	-0.07642**	2.60%	-0.07635**	2.78%
TaxRate	-0.0033	89.56%	-0.00506	84.02%	0.00051	98.37%
IndexReturn	0.00273	31.26%	0.00241	37.30%	0.00224	41.18%
IndexVolatility	-0.0009	84.59%	-0.00044	92.47%	-0.00021	96.34%
Year 2002	0.00022	94.07%	0.00088	76.95%	0.00084	78.10%
Year 2003	-0.0003	91.69%	0.00020	94.72%	0.00061	84.57%
Year 2004	-0.0017	57.88%	-0.00170	58.01%	-0.00129	67.49%
Year 2005	-0.0008	81.11%	-0.00074	81.62%	-0.00068	83.00%
Year 2006	-0.0019	53.98%	-0.00198	53.29%	-0.00194	54.39%
Year 2007	-0.0003	90.75%	-0.00047	87.50%	-0.00050	86.86%
Year 2008	-0.0000	99.99%	-0.00038	91.31%	-0.00064	85.74%
Year 2009	0.00033	92.50%	0.00101	77.59%	0.00087	80.65%
Year 2010	0.00048	87.63%	0.00055	85.86%	0.00084	78.53%
Year 2011	-0.0017	56.66%	-0.00112	70.69%	-0.00121	68.67%
Year 2012	-0.0000	98.82%	0.00037	90.78%	0.00086	78.93%
Year 2013	-0.0002	93.75%	0.00007	98.24%	0.00017	95.84%
Year 2014	-0.0036	23.91%	-0.00305	32.00%	-0.00290	34.61%
Year 2015	-0.0015	63.17%	-0.00134	67.18%	-0.00078	80.57%
Year 2016	-0.0046	14.85%	-0.00449	15.98%	-0.00419	19.19%
Year 2017	-0.005	13.18%	-0.00472	15.48%	-0.00438	18.94%
Year 2018	-0.0042	21.01%	-0.00415	21.26%	-0.00371	26.74%

* Implies significant at 10%.

** Implies are significant at 5%.

*** Implies significant at 1%.

**Table 3:** Leniency of personal bankruptcy and self-employment–adjusted group with different time lags.

	Adjusted Group		Adjusted Group			Adjusted Group	
	(N = 172, *R*^2^ = 24%)		(N = 172, *R*^2^ = 22%)			(N = 244, *R*^2^ = 21%)	
	Time lag: -1		Time lag: 0			Time lag: +1	
	Beta	p value	Beta		p value	Beta	p value
Leniency index	0.00515***	0.80%	0.00400**	3.33%		0.00323*	8.77%
Unemployment rate	-0.00360	84.19%	-0.00548	76.71%		-0.00553	76.96%
R&D	-0.07029	82.13%	-0.14209	64.98%		-0.18454	55.89%
FemLaborForce	0.09744**	4.66%	0.09834**	4.68%		0.10186**	4.05%
GDP_growth_rate	-0.03724*	5.56*%	-0.03619*	6.62%		-0.03840*	5.21%
Gini Index	-0.08561*	7.20*%	-0.07976*	9.56%		-0.07903	10.12%
TaxRate	0.01499	65.39%	0.01290	70.31%		0.01880	57.93%
IndexReturn	0.00277	44.02%	0.00237	51.28%		0.00194	59.75%
IndexVolatility	0.00410	70.99%	0.00430	69.90%		0.00369	74.22%
Year 2002	-0.00536	28.50%	-0.00389	43.90%		-0.00400	42.93%
Year 2003	-0.00557	24.84%	-0.00434	37.14%		-0.00378	44.00%
Year 2004	-0.00683	15.32%	-0.00650	17.75%		-0.00586	22.59%
Year 2005	-0.00672	16.83%	-0.00636	19.59%		-0.00625	20.67%
Year 2006	-0.00913*	6.60%	-0.00871*	8.15%		-0.00858*	8.82%
Year 2007	-0.00581	24.98%	-0.00542	28.70%		-0.00538	29.40%
Year 2008	-0.00643	27.95%	-0.00629	29.41%		-0.00645	28.65%
Year 2009	-0.00849	13.46%	-0.00697	22.06%		-0.00679	23.58%
Year 2010	-0.00587	26.77%	-0.00508	34.02%		-0.00448	40.31%
Year 2011	-0.01074*	5.46%	-0.00901	10.52%		-0.00890	11.17%
Year 2012	-0.00721	20.05%	-0.00591	29.43%		-0.00497	37.83%
Year 2013	-0.00727	18.71%	-0.00623	25.95%		-0.00588	28.93%
Year 2014	-0.01226**	2.99%	-0.01053*	6.03%		-0.01031*	6.76%
Year 2015	-0.01108	5.09%	-0.00978*	8.40%		-0.00882	11.80%
Year 2016	-0.01364**	1.68%	-0.01239**	2.96%		-0.01185**	3.81%
Year 2017	-0.01307**	2.88%	-0.01146	5.35%		-0.01088	6.78%
Year 2018	-0.01245**	4.91%	-0.01115	7.79%		-0.01042	10.03%

* Implies significant at 10%.

** Implies significant at 5%.

*** Implies significant at 1%.

To investigate the robustness of our estimates, we repeat our calculation with the subsample of the Adjusted Group, where countries with limited eligibility for entrepreneurs are excluded. These results are reported in Table 3. Again, the coefficient of the leniency index is significant with no time-lag, furthermore, it is highly significant in the case of a time-lag of –1. The results show even lower significance in the case of a time-lag of +1 year.

To further check the robustness, we also run our calculation with a negative time-lag of two years (*T–2*). The coefficient of the Leniency index does not prove to be significant in this case. In almost all the cases the same control variables become significant (“GDP growth rate,” “Gini Index” and “Female labour force”) with consistent beta signs.

The beta coefficients also imply the magnitude of the change in the number of entrepreneurs due to changes in the leniency of the regulation. Our results suggest that if the leniency index changes by 1.0 the stagnated self-employment rate (with no increase or decrease) increases by 0.4 to 0.5% in one year. If self-employment rates do not stagnate, the change in the leniency index speeds up or slows down the change in self-employment by the same magnitude.

As for the limitation of our research, due to access to data and comparability, we examine only the formal aspects of the institutional and regulatory environment and their effect on self-employment. In addition, our control variables are economic-social determinants; we do not consider any informal aspects of the institutional environment and neither psychological, cultural, or political factors. We must consider that reforming bankruptcy laws is not the unique solution to spur entrepreneurship, but a development of institutional environment is also necessary.

We use self-employment for measuring entrepreneurship, which may not fully capture all entrepreneurial dynamics; although, it is one of the most used proxies for entrepreneurial development. Despite the use of single data sources (World Bank, Eurostat), the exact definitions and coverage of our variables may vary from country to country. Additionally, data collection methods and timing of the underlying surveys can also differ. On the other hand, we choose data sources where all variables are available and comparable for all countries in the scope of the research.

We must also consider the limitation of Walter and Krenchel [7] where the scoring of the leniency index variables after reforms is based on the interpretation of legislative formulations, which may be affected by differences between the case law and the verbatim legal text. Identifying changes in scores for some indicators can cause some uncertainty even if the scoring is assessed by local legal experts.

Like other similar research [9], we do not measure the credit rational impact on markets, that is, how changes in the bankruptcy system may affect the supply and condition of credit. Leniency reforms might induce opposite effects and lead to tightening access to credit for small businesses and reducing entrepreneurial developments.

The data, results, and conclusions are for the EU countries and cannot be directly applied to countries on other continents. However, the countries studied are very heterogeneous in terms of legal systems, legal origin, development, and the dynamic of rate of self-employment, so the results are likely to be applicable for other developed countries with similar characteristics.

Finally, as regards the interpretation of these results, it should also be noted that we do not know how long the 0.4–0.5% increase in the growth rate of the self-employment rate will last. This long-term effect could be a subject of further research.

## Discussion and conclusions

With the help of the composite leniency index, as a novelty, we map out how personal bankruptcy law varied and changed across Europe from 2000 to 2019. We conclude that there were many types of reforms touching different elements of the legislations, and regulations were mostly shifted onto a more lenient level. As a contribution to an institution-based view of entrepreneurship, we measure the leniency and changes in the personal bankruptcy systems of the EU countries in a complex way and analyze how it stimulated entrepreneurship developments across these countries.

The results support our hypothesis that there is a positive association between self-employment rates and the leniency in the EU countries by measuring the leniency with a time-varying composite index. These results of significantly positive associations support the findings of several other papers presented in previous sections [9, 14, 16, 18, 29–32]). These studies also found positive associations between the characteristics of leniency and entrepreneurialism although using different approaches. While most of the papers focused on the US market and the characteristics of homestead exemptions of the states or the BAPCPA reform, we examine 24 EU countries of very different systems and measure the consequences of new implementations, reforms, and changes of 20 years. The few research studies that measured leniency with some selected variables also found positive association [9, 32] but with limitations. Lee et al. [32] examined general bankruptcy law while Armour and Cumming [9] personal bankruptcy systems with similar variables, but these indicators–some, mainly “discharge features” of the systems–were not time-varying. They also highlighted that people might not realize and react on nuances of bankruptcy laws when they decided to start entrepreneurship. Our study does not have these limitations. In contrast, as we measure leniency with a composite index over time, we incorporate time-varying bankruptcy variables. We are able to account for the development in regulations during periods and countries and capture the changes in personal bankruptcy regulations among countries over time. Furthermore, we consider not just one or two elements, nuances in the legislations, but we measure and consider the complex change in the system in many dimensions.

Besides underpinning the results of former studies, we can also show several novelties and explain the findings of other papers. The changes in the leniency index that we explore also explain why Fossen and König [40] contradicted the former results and found no evidence of a positive effect. The referred German reform only slightly changed the leniency index (Fig 1) and could not influence the German entrepreneurial market. Our analysis is new and improved in the sense that similar study of Armour and Cumming [9] included countries (France, Belgium) in the regression where there existed eligibility constraints for entrepreneurs–and these constraints cast doubt on the incentive for starting entrepreneurship. When we exclude these countries from our analysis, our results present a more significant association.

Our results also show that there is a time-lag in the association. Although we would expect a positive time-lag, as was examined in the research of Lee et al. [32], somewhat surprisingly, our findings indicate that results are more significant considering a one-year negative time-lag. It shows that more entrepreneurs enter the market even in the parliamentary debate phase, a year before the law enters into force.

Considering policy implications our results show that policy makers can encourage entrepreneurship by materially changing the different elements, and thus the debtor friendliness of personal bankruptcy systems. Our calculations also imply the magnitude, the necessary extent, of a reform that is required to achieve material changes in the number of entrepreneurs. If the leniency index changes by +1.0, the stagnated self-employment rate increases by 0.4 to 0.5%. Translated to practical policy implication, a growth of 1.0 in the leniency index corresponds to the introduction of a new, relatively conservative regulation or a significant reform of a conservative regime to a very lenient level. This effect is of the same order of magnitude as other types of reforms and measures to influence entrepreneurial behavior presented in the literature review section, the effect of health insurance premiums examined by Heim and Lurie [10] or financial liberalization studied by Jha and Bhuyan [11].

Our panel regression results show that these changes or even the anticipation of lenient changes positively affect the self-employment rates with high significance in the EU countries. The finding of more significant association with a one-year negative time-lag also has important policy implications. It shows that the strong anticipation of the draft law, the policy proposal for a more lenient system can immediately change the risk-reward profile and influence entrepreneurship even before the implementation of the actual reform.

As discussed above, we do not know how long the increase in the growth rate of the self-employment rate will last, which necessitates further in-depth research. Further research may also explore the background of how the preparation of the law and the legislative process leads to an increase in self-employment. Further studies could explore how the leniency index developed in other countries outside the EU and whether associations with entrepreneurship based on the leniency index changes could be detected. Finally, the risk-reward profile cannot only be analyzed with the rate or number of new entrepreneurs stepping in, but how their risk appetites in operation, investment, and financing possibly change, which analysis can also be the basis for further research.

## Supporting information

S1 Fig Self-employment developments–group of countries with zero trend. (TIF)

S2 Fig Self-employment developments–group of countries with negative trend. (TIF)

S3 Fig Self-employment developments–group of countries with positive trend. (TIF)

S1 Table Definition and sources of variables. (DOCX)

S2 Table Definition of variables in the leniency index. (DOCX)
