# Supplementary figures and images for: How does the leniency of personal bankruptcy law affect entrepreneurship in EU countries?
Source: PLoS One. 2022 Jul 28;17(7):e0272025. doi: 10.1371/journal.pone.0272025 (PMC9333209; doi:10.1371/journal.pone.0272025)

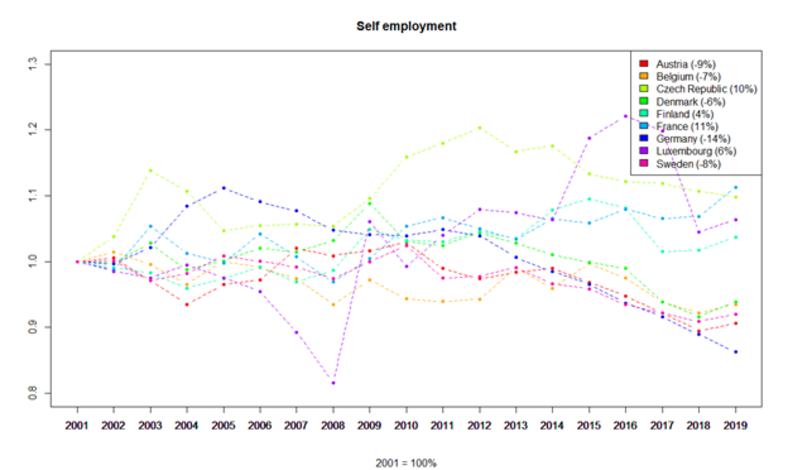

Supplement: S1 Fig — (TIF) [file pone.0272025.s001.tif]

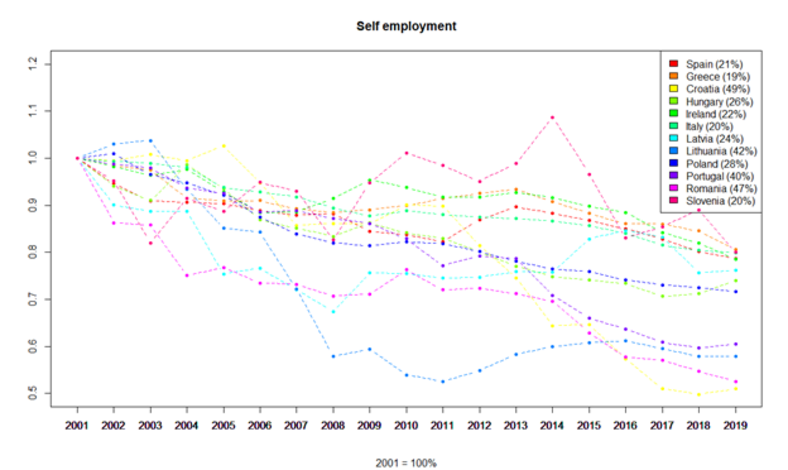

Supplement: S2 Fig — (TIF) [file pone.0272025.s002.tif]

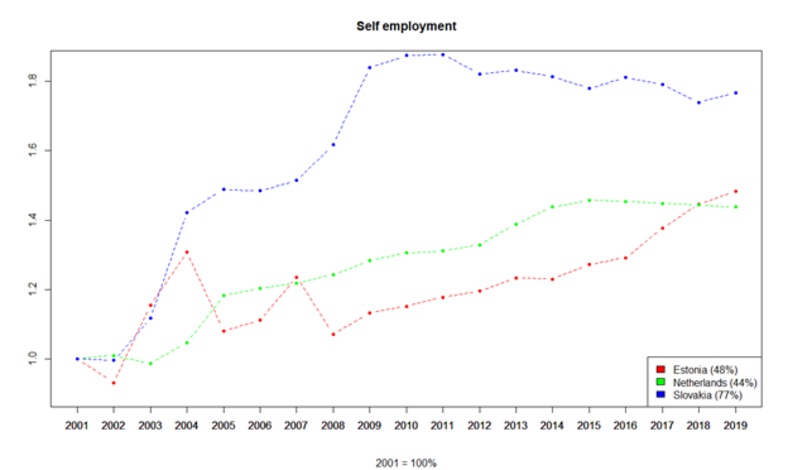

Supplement: S3 Fig — (TIF) [file pone.0272025.s003.tif]
