# Supplementary material for: How does the leniency of personal bankruptcy law affect entrepreneurship in EU countries?
Source: PLoS One. 2022 Jul 28;17(7):e0272025. doi: 10.1371/journal.pone.0272025 (PMC9333209; doi:10.1371/journal.pone.0272025)
Supplement: S1 Table — (DOCX) [file pone.0272025.s004.docx]

| **World Bank-GDP annual growth rate** | Source: https://data.worldbank.org/indicator/NY.GDP.MKTP.KD.ZG The annual percentage growth rate of GDP at market prices is based on constant local currency. Aggregates are based on constant 2010 U.S. dollars. GDP is the sum of gross value added by all resident producers in the economy plus any product taxes and minus any subsidies not included in the value of the products. It is calculated without making deductions for depreciation of fabricated assets or depletion and degradation of natural resources.  Periodicity: Annual  Unit of measurement: percentage |
| --- | --- |
| **Market Indices and volatilities** | Source: Reuters, yahoo finance, stock exchange homepages  Periodicity: Annual  Unit of measurement: percentage |
| **World Bank - Gini_Inequality** | Source: https://data.worldbank.org/indicator/SI.POV.GINI  Gini index measures the extent to which the distribution of income (or, in some cases, consumption expenditure) among individuals or households within an economy deviates from a perfectly equal distribution. A Lorenz curve plots the cumulative percentages of total income received against the cumulative number of recipients, starting with the poorest individual or household. The Gini index measures the area between the Lorenz curve and a hypothetical line of absolute equality, expressed as a percentage of the maximum area under the line. Thus, a Gini index of 0 represents perfect equality, while an index of 100 implies perfect inequality.  Periodicity: Annual  Unit of measurement: percentage |
| **World Bank_Labour force participation rate, female (% of female population ages 15+) (modelled ILO estimate)** | Source: https://data.worldbank.org/indicator/SL.TLF.CACT.FE.ZS  Labour force participation rate is the proportion of the population aged 15 and older that is economically active: all people who supply labour for the production of goods and services during a specified period.  Periodicity: Annual  Unit of measurement: percentage |
| **World Bank_Gross domestic expenditure on research and development** | Source: https://data.worldbank.org/indicator/GB.XPD.RSDV.GD.ZS  Gross domestic expenditures on research and development (R&D), are expressed as a per cent of GDP. They include both capital and current expenditures in the four main sectors: Business enterprise, Government, Higher education and Private non-profit. R&D covers basic research, applied research and experimental development.  Periodicity: Annual  Unit of measurement: percentage |
| **World Bank - Unemployment, total (% of the total labour force) (modelled ILO estimate)** | Source: https://data.worldbank.org/indicator/SL.UEM.TOTL.ZS Unemployment refers to the share of the labour force that is without work but available for and seeking employment.  Periodicity: Annual  Unit of measurement: percentage |
| **Eurostat - Tax rate** | Source: https://ec.europa.eu/eurostat/databrowser/view/EARN_NT_TAXRATE__custom_1131537/default/table?lang=en;  Tax rate, defined as the income tax on gross wage earnings plus the employee's social security contributions less universal cash benefits, expressed as a percentage of gross wage earnings;  Periodicity: Annual  Unit of measurement: percentage |
