# Supplementary material for: How does the leniency of personal bankruptcy law affect entrepreneurship in EU countries?
Source: PLoS One. 2022 Jul 28;17(7):e0272025. doi: 10.1371/journal.pone.0272025 (PMC9333209; doi:10.1371/journal.pone.0272025)
Supplement: S2 Table — (DOCX) [file pone.0272025.s005.docx]

| **Dimensions** | **Indicators*** **in the leniency index**** |
| --- | --- |
| 1. *Straight bankruptcy* | - Straight bankruptcy - Walk-away opportunity |
| 1. *Eligibility*: | - Entitled persons to participate - Preconditions, constraints - Exclusion criteria - Preconditions in debt - Stigmas in eligibility |
| 1. *Expensiveness* | - Starting costs - Distribution of costs - Deposit requirements |
| 1. *Complexity* | - Types of creditors - Officers who conduct, regime types - Complexity to start - Complexity of the process - Debt counselling service |
| 1. *Process* | - Pre-action stage - Initiator of the procedure - Initiator of the first draft of the repayment plan - Creditors included - Degree of disability of the debtor - Decision mechanism - Asset sale - Possible consequences of commencement - Exemptions - Possible easing measures - Possible penalties |
| 1. *Conditions for discharge* | - Existence of a full discharge - Length of repayment obligation, - Level of necessary repayment - Conditions of discharge - The validity of discharge |
| 1. *Stigmas* | - Other provisions against the debtor - Publicity stigmas - Restrictions - Stigmatic names |

* Categorical scale: 0-1-2 (from less to more lenient)

** Leniency Index applies equal weights (EW) with linear aggregation within one dimension and budget allocation process (BAP) and linear aggregation for calculating the final index score from the seven main dimensions. Final index scores are between 0 to 2. Adjusted with annual periodicity. See description of indicators and methodology more in detail in Walter and Krenchel [7]
